# Designing and Synthesis of New Isatin Derivatives as Potential CDK2 Inhibitors

**DOI:** 10.3390/ijms23148046

**Published:** 2022-07-21

**Authors:** Przemysław Czeleń, Agnieszka Skotnicka, Beata Szefler

**Affiliations:** 1 Department of Physical Chemistry, Faculty of Pharmacy, Collegium Medicum, Nicolaus Copernicus University, Kurpinskiego 5, 85-096 Bydgoszcz, Poland; beatas@cm.umk.pl; 2 Faculty of Chemical Technology and Engineering, Bydgoszcz University of Science and Technology, Seminaryjna 3, 85-326 Bydgoszcz, Poland; askot@pbs.edu.pl

**Keywords:** isatin, CDK2, competitive inhibition, molecular dynamics, synthesis, spectroscopic properties

## Abstract

Tumors are still one of the main causes of death; therefore, the search for new therapeutic agents that will enable the implementation of effective treatment is a significant challenge for modern pharmacy. One of the important factors contributing to the development of neoplastic diseases is the overexpression of enzymes responsible for the regulation of cell division processes such as cyclin-dependent kinases. Numerous studies and examples of already-developed drugs confirm that isatin is a convenient basis for the development of new groups of inhibitors for this class of enzyme. Therefore, in this work, a new group of potential inhibitors of the CDK2 enzyme, utilizing isatin derivatives and substituted benzoylhydrazines, has been designed based on the application of computational chemistry methods, such as docking and molecular dynamics, and their inhibiting ability was assessed. In the cases of the selected compounds, a synthesis method was developed, and the selected physicochemical properties of the newly synthesized derivatives were estimated. As part of the completed project, new compounds are developed which are potential inhibitors of the CDK2 enzyme.

## 1. Introduction

**Figure 1:**
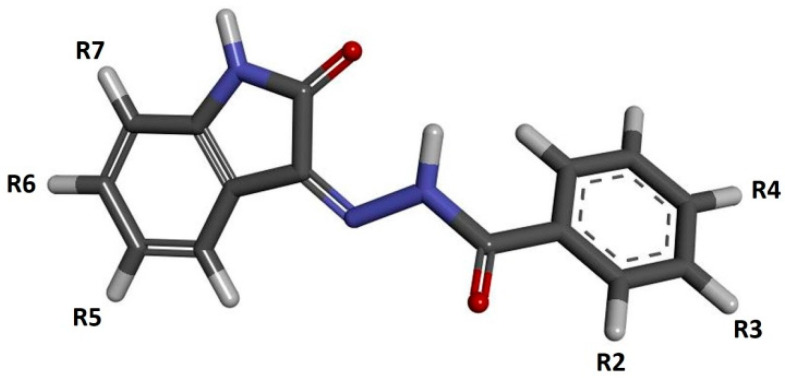
The graphic representation of isatin derivatives. Symbols R2–R7 represent places of addition of new chemical residues modifying the chemical properties of the native compound.

Isatin (1H-indol-2,3-dione) is a chemical compound which, as natural alkaloid, may be extracted from plants of the Isatis genus, occurring all over the world at various latitudes [1,2,3]. The significant chemical and biological activities of isatin meant that this compound and its numerous derivatives, obtained from natural products, have widely been used in medicine for many centuries [4,5]. In terms of the pharmaceutical activity of such compounds, they have anti-inflammatory, antiviral, antibacterial, antifungal, anticonvulsant, anxiogenic and anticancer properties [6,7,8,9,10]. The large spectra of pharmacological activities of isatin derivatives are associated with the significant chemical variety of these compounds, within which one can distinguish hydrazones, thiosemicarbazones, oximes, spiro-oxindoles, imines and many more. The common element of this broad group of compounds is the oxindole system, which is the core repeatedly used in the development of new groups of competitive inhibitors [11,12,13,14,15,16,17,18,19,20,21]. The anticancer activity of new drugs is mainly related to the competitive inhibition of biological targets, the overexpression of which drives the accelerated growth of cancer cells. The group of enzymes associated with the development of cancers includes the set of SER/THR kinases classified as cyclin-dependent kinases (CDKs), especially CDK2. This enzyme plays a crucial role in the regulation of the cell cycle. The CDK2 and other cyclin-dependent kinases are also crucially important in the regulation of the other enzymes involved in transcription and replication processes [22,23,24]. A significant group of CDK2 inhibitors is isatin derivatives and its analogs, which contain a characteristic oxindole core in their structure. Numerous studies show that this type of compound has a notable affinity for the active site of this enzyme [12,16,25]. The location of the donors and acceptors of hydrogen bonds in the oxindol core ensures the possibility of creating stable interactions in the hinge region with aminoacids such as LEU83 and GLU81 [16,25]. The presence of additional hydrogen bond acceptors in oxindolic systems may provide an opportunity to create interactions with LYS33 and ASP145 [15,26,27]. Based on these guidelines, this work will develop a new group of isatin derivatives based on the reaction of isatin derivatives with the set of substituted benzoylhydrazines. The schematic representation of the isatin-based benzoylhydrazine structure is presented in Figure 1. During the creation of new potential inhibitors, the impact of the presence of various substituents (positions R2–R7) on their binding activity with the CDK2 active site will be evaluated.

## 2. Results and Discussion

### 2.1. Design and Computational Analysis of Binding Activity

The isatin derivatives were created in two stages. Firstly, the modification of the isatin molecule was chosen. Based on the commercially available isatine derivatives containing substituents localized in positions 5, 6 and 7, model structures of inhibitors obtained by reaction with non-substituted benzoylhydrazide were created. The binding affinity of such molecules towards cyclin dependent kinase was evaluated using docking methods.

**Figure 2:**
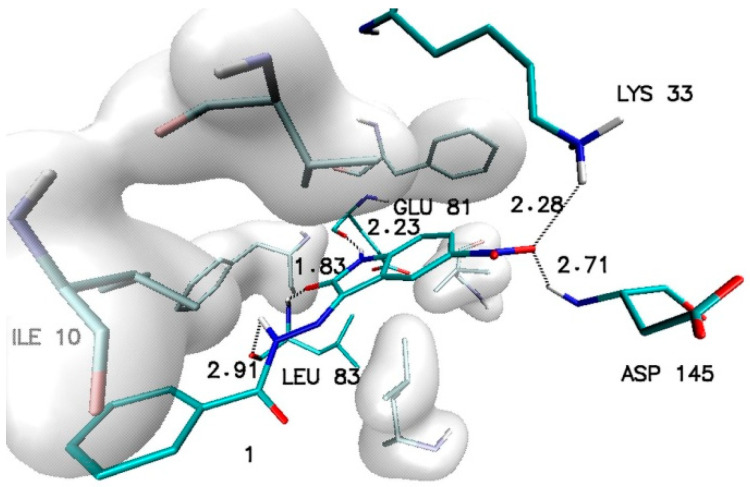
The graphic representation of interactions involved in stabilization of CDK2 complex with *N*′-[5-nitro-2-oxo-1,2-dihydro-3*H*-indol-3-ylidene]benzohydrazide (**1**).

The outcome of these calculations is presented in Table 1. Considering all of the collected data, it can be observed that the least effective are modifications located in position 7, while the most favorable effects involving an increase in affinity are observed in the case of modifications located in positions 5 and 6. Among all of the considered structures, the highest value of affinity was recorded for the 5-nitroisatin derivative. The structure of the complex created by this molecule with the CDK2 active site is presented in Figure 2.

**Figure 3:**
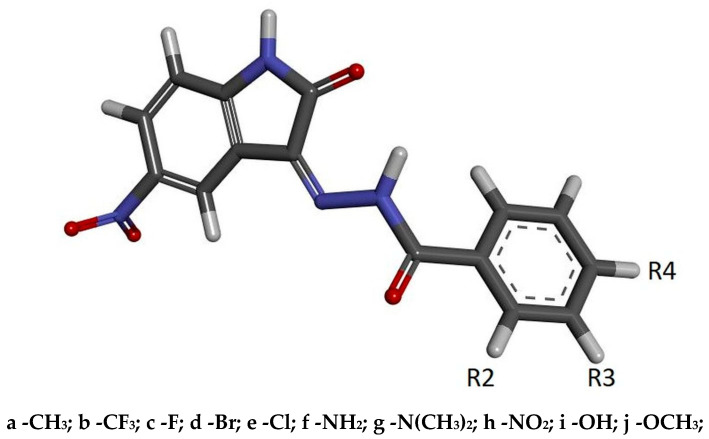
The graphic representation of 5-nitroisatin-based benzoylhydrazines. The markings R2, R3 and R4 represent the places of chemical group substitution. The derivative markings consist of a number symbolizing the place of substitution and the letter assigned for a specific chemical group.

The stability of such a system is maintained by a network of interactions including hydrogen bonds and hydrophobic interactions. Besides the hydrogen bonds created by atoms from a molecular core with GLU81 and LEU83, the activity of oxygen atoms from the nitro group was also observed, which participates in interactions with LYS33 and ASP145. Based on the values of binding affinity and the presence of additional binding factors, related with the existence of two additional hydrogen bond acceptors, 5-nitroisatin was chosen for the next stage of research. Based on commercially available substituted benzoylhydrazide derivatives, 30 new potential CDK2 inhibitors were created; the scheme of the foreseen modifications of the native molecule are presented in Figure 3. In the nomenclature adopted in this work, a native molecule based on unmodified benzoylhydrazide was marked with the number “1”, while in the case of subsequent derivatives, the designation consists of a number symbolizing the place of substitution and the letter assigned for a specific chemical group. The binding capabilities towards the CDK2 active site of each newly created molecule were evaluated using docking methods. Table 2 presents the values of binding affinity and the inhibition constants obtained for all 5-nitroisatin-based benzoylhydrazines.

The inhibition constant (IC) for ligand molecules was estimated based on the Van ’t Hoff isotherm equation.

$$K_{I}=exp^{\left(\frac{\Delta G_{b}}{\mathrm{RT}}\right)}$$

Comparing the values obtained for new molecules with the native one, it can be observed that the introduction of modifications in the aromatic system of the considered derivatives does not always increase their binding capacities and, in some cases, even lowers them. It is especially visible in the case of modifications located in the para position (R4), since none of the newly generated molecules with such modification demonstrated a noticeable increase in binding activity.

**Figure 4:**
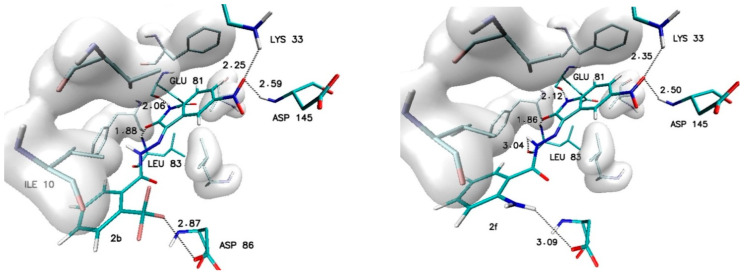

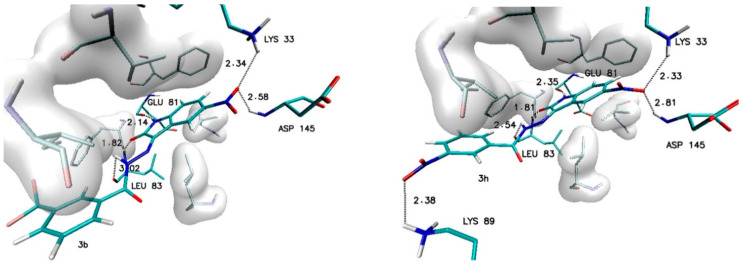
The graphic representation of interactions involved in stabilization of CDK2 complex with chosen 5-nitroisatin-based benzoylhydrazines.

Different observations can be made in the case of modifications present in the ortho (R2) and meta (R3) positions. Among all considered substituents, the greatest influence on the binding capability was observed in the case of the trifluoromethyl group, the appearance of which caused the greatest increase in affinity value (−0.5 kcal/mol) and the most convenient value of the inhibition constant (46.8 nM). An analogous increase was also recorded for the methyl group located in the meta position (R3). In the case of the other considered substituents, the observed increases in affinity ranged from −0.2 to −0.3 kcal/mol. For all complexes formed by derivatives with an increase in affinity of at least −0.2 kcal/mol, structural stability was analyzed using molecular dynamics methods. The structures of the chosen complexes are presented in Figure 4 and Figure S1 (Supplementary Materials). Such systems are maintained by a network of numerous interactions of different types. The most important hydrogen bonds identified in all the analyzed systems are summarized in Table 3. Each considered molecule creates bindings with aminoacids such as LYS33, GLU81, LEU83 and ASP145. Based on the geometric hydrogen bond strength classification, interactions created with GLU81, LEU83 and LYS33 can be qualified as medium-strength hydrogen bonds, while interactions with ASP145 are classed weak bonds. In the case of molecules such as 2b, 2f and 3h, interactions created by atoms from chemical groups located in benzoylhydrazide part of the molecule were also observed.

The structural stability of considered complexes and the time evolution of such systems was evaluated based on an analysis of root mean square deviation (RMSD) values and structural descriptors, defining the interactions involved in complex stabilization such as distances and angles measured for hydrogen bond donors and acceptors identified for particular hydrogen bond. Figure S2 (Supplementary Materials) presents distributions of the RMSD values estimated for inhibitors and CDK2 protein. In each case, values were calculated with regard to the starting point, which was the geometry of the complex obtained during docking stage.

An analysis of such distributions and averaged values is presented in Table 4; this shows that not all proposed derivatives have the ability to create stable complexes with the active place of the considered protein. In a few cases, such as molecules 2c, 2e, 3c and 3d, large fluctuations in RMSD values are observed, indicating the noticeable conformational instability of such molecules in the space of a CDK2 active site. The inability to maintain the original conformation obtained during docking or to adopt a new one ensuring a better fit to the active place may indicate insufficient matching, which is caused by the presence of a possible steric hindrance in the considered molecules. Completely different characteristics of the discussed properties are shown by molecules such as 1, 2b, 2d, 3b, 3e, 3f and 3h, for which uniform distributions and low standard deviation for the analyzed populations of RMSD values indicate the conformational stability of the analyzed systems.

An important factor for assessing the stability of the complexes formed by potential inhibitors with the active site of a biological target is the evaluation of the durability of the interactions responsible for its maintenance. During the docking stage, the primary structures of the complexes were created, in which interactions such as hydrogen and halogen bonds were identified. Table 5 and Table S1 (Supplementary Materials) contain a list of the most important impacts that showed sufficient durability to exclude their incidental occurrence. Among all of the considered interactions, the most important and the most stable in each of the complexes are hydrogen bonds formed by the oxygen and the hydrogen atoms of the isatin core with LEU83 and GLU81. The presence of these hydrogen bonds was confirmed in 100% of conformers accumulated during molecular dynamics for each of the analyzed complexes. The observed differences between particular systems are visible in the distribution of the bond lengths dominant within the compared populations. In the case of bonds created with GLU81, over 90% of tested conformers creates at least medium-strength hydrogen bonds (distance ≤ 2 Å), the exceptions are only the complexes created by 2c and 3c molecules. The distances measured for hydrogen bonds created with LEU83 exhibit much larger diversity—in this case, there is at least a 70% share of medium-strength hydrogen bonds observed only for conformers from complexes created by 1, 2d, 2e, 2f, 3b, 3e and 3f molecules. The activity of oxygen atoms from the nitro group localized in the isatin core and the significance of hydrogen bonds created by them is diametrically different for complexes created by individual derivatives. Among the analyzed systems, one can distinguish complexes where the bonds with ASP145 almost disappeared and those created by LYS33 have been significantly reduced, as in the case of 2c and 3c complexes. A group of complexes is also noticeable where, for at least 80% of the conformers, stable bonds with at least one of the amino acids are observed, for example, complexes formed by the molecules 1, 2d, 2f, 3b, 3e, and 3h. The highest binding activity for the nitro group was recorded in the case of derivative 2b, in which stable hydrogen bonds were observed both for the ASP145 and LYS33 in over 90% of the analyzed conformers. Based on the structures of complexes obtained during docking for many of the derivatives considered, the possibility of creating a second hydrogen bond with LEU83 was expected. An analysis of the results of molecular dynamics simulation showed the existence of stable and numerous interactions of this type only for complexes created by 2b (85% of conformers) and 3f (98.9% of conformers) derivatives. An analysis of the interactions in the complex formed by the derivative 2f shows the presence of a halogen bond between the fluorine atoms of the 3-fluoromethyl group and the oxygen atom of ILE10. The distance between the donor and acceptor of this interaction in the scope of the entire simulation ranges from 2.6 to 3.2 Å. The presence of such an interaction contributes to the overall stabilizing effect of the analyzed complex.

**Figure 5:**
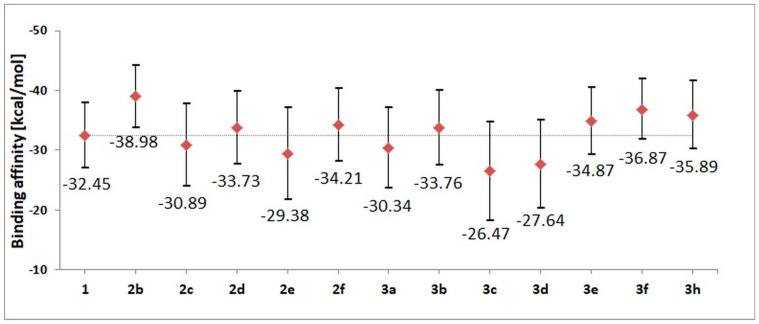
The values of binding enthalpy (kcal/mol) estimated for all complexes considered during molecular dynamics stage.

The stability of complexes was also evaluated using the molecular mechanics Poisson–Boltzmann surface area (MMPBSA) method, which helped to evaluate the values of binding affinity characterizing particular complexes. Based on the conformers from the last 60 ns of molecular dynamics simulation, enthalpic contributions to the binding affinity were estimated. The obtained values are presented in Figure 5; numeric labels represent accurate values of affinity, while the graphic markers correspond to a spread of values, taking into account the standard deviation. Based on the gathered values, the largest affinity toward the active site of CDK2 is shown by 2b, 3f and 3h derivatives. The energy characteristics of the systems corroborate the previous structural observations based on an analysis of the conformational properties of ligands and the stability of interactions involved in the maintenance of the complexes. Based on the summary characteristics of the analyzed systems, for the experimental phase, including the synthesis and characterization of molecular and spectroscopic properties, derivatives 1, 2b, 2d, 2f, 3b, 3e, 3f and 3h were selected.

### 2.2. Synthesis and NMR Data

**Scheme 1:**
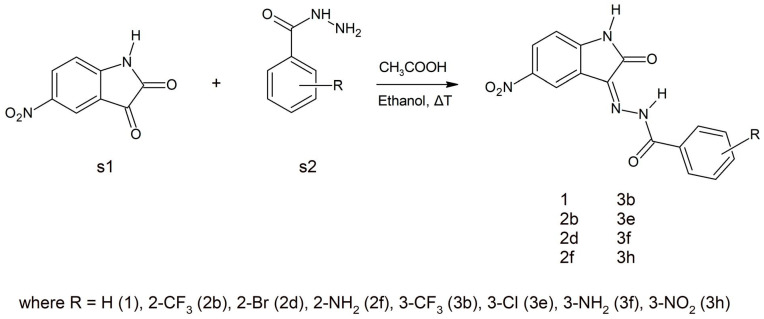
A schematic representation of the synthesis of the selected 5-nitroisatin-based benzoylhydrazines.

Various synthetic procedures for 5-nitroisatin-based benzoylhydrazines can be found in the literature [28,29,30]. The simplest indicates that 5-nitroisatin (s1) is heated with substituted benzoylhydrazine (s2) in ethanol in the presence of acetic acid (Scheme 1). All the derivatives were analyzed using IR, NMR and also elemental analysis.

Structurally, the synthesized 5-nitroisatin-based benzoylhydrazines can exist in *cis*- and *trans*-isoforms. Although all of the derivatives demonstrated the presence of both isomers in the solution at room temperature, with the *cis*-isomer being the predominant one. It should be noted that in all cases, their ^1^H NMR spectra showed a more intense signals for hydrogen atoms of *cis*-isomer. Moreover, the calculated energies showed that more stable are *cis-*isomers (Table 6).

**Figure 6:**
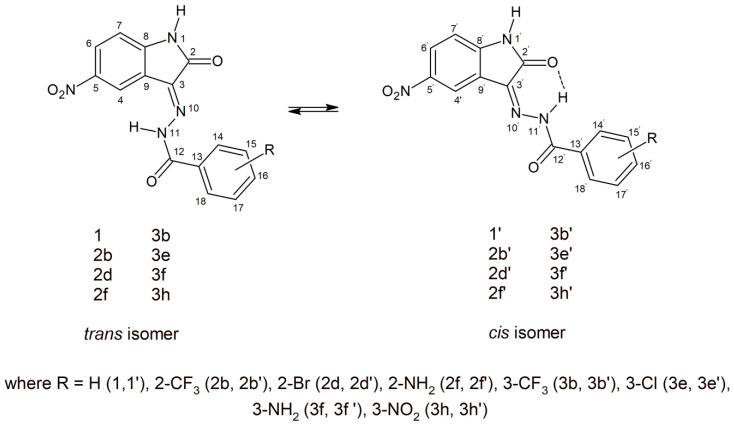
Possible geometry of the synthesized 5-nitroisatin-based benzoylhydrazines.

As shown in Figure 6, in the chemical shift structure of the *cis* form, there is a possibility of intramolecular hydrogen bonding between the hydrogen atom of the hydrazone section and the oxygen atom of the adjacent carbonyl group [31]. In addition, in derivatives substituted in position 2 (2b, 2d and 2f), the presence of an intramolecular hydrogen bond between the substituent and the carbonyl oxygen of benzoylhydrazine was observed. According to R.S. Hunoor et al. [32], the N–H group (hydrazine part) is sandwiched between carbonyl (C=O) and N=C azometine nitrogen and intramolecular hydrogen bonding with carbonyl oxygen of 5-nitroisatin. All these interactions make it difficult to unambiguously assign aromatic hydrogens and carbons to individual isomers of 2-substitued 5-nitroisatin-based benzoylhydrazines.

**Figure 7:**
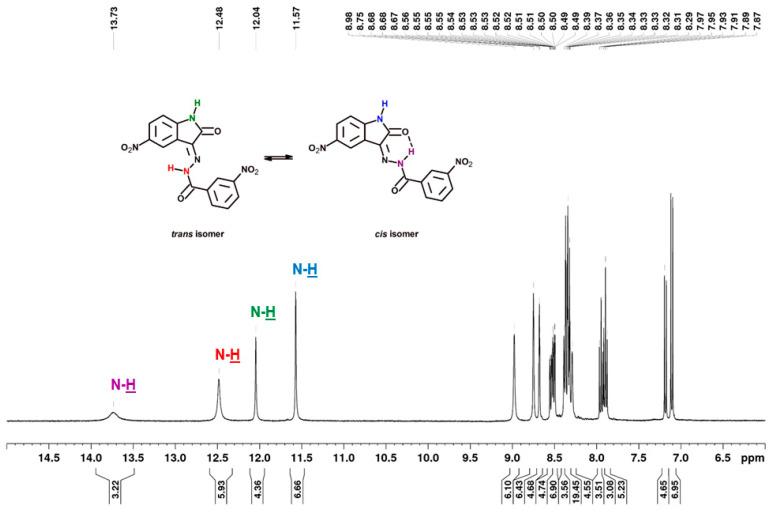
^1^H NMR spectrum (400 MHz) of 3-nitro-*N*′-[5-nitro-2-oxo-1,2-dihydro-3*H*-indol-3-ylidene]benzohydrazide (3h) in DMSO-d_6_.

As previously mentioned, the compounds showed in Figure 6 were present in solution as *trans-* and *cis*-isomers. In all cases, their ^1^H NMR spectra showed a signal with chemical shift in the range of 13.16–13.37 ppm, corresponding to the N–H of benzoylhydrazine involved in the relevant intramolecular hydrogen bond for *cis*-isomers. For the same proton the *trans*-isomer (without intramolecular hydrogen bond), the signal was observed in the range of 12.02–12.88 ppm. The ^1^H-NMR signal of N–H of isatin can be seen at δ = 11.51–11.57 ppm and δ = 11.96–12.04 ppm in DMSO, which is a singlet, for *cis-* and *trans*-isomers, respectively (Figure 7). The first signal observed with the least chemical shift in aromatic protons relates to the H-7 and H-7′ of isatin, which appears as a doublet signal in the region of 6.85 to 7.18 ppm (*J* = 8.56–8.86 Hz). The next protons of isatin (H-4, H-4′ and H-6, H-6′) appeared in the region of 8.23 to 9.06 ppm. The aroyl (hydrazone) part of the target compounds showed appropriate signals in the range of 6.67–8.18 ppm, depending on the type of substituent. The carbon signals of the most characteristic carbon atoms, such as carbonyl carbon C-12/C-12′ and lactonyl carbon C-2/C-2′, were observed at ca. 167 and 164 ppm, respectively. The azomethine carbon C-3/C-3′ at ca. 137 ppm was not always recorded.

### 2.3. Infrared Spectral Studies

Vibrational spectroscopy is used extensively in the study of molecular conformations, the identification of functional groups and reaction kinetics, etc. Infrared spectral data for the synthesized 5-nitroisatin-based benzoylhydrazines are presented in Table 7. Sharp bands of medium intensity at 3167–3303 cm^−1^ in the analyzed compounds spectrums were due to stretching vibrations of the –NH group. The absorption band characteristic of ν(C=O) vibrations of isatin and benzoylhydrazine fragments appeared in the range 1697–1751 cm^−1^ and 1622–1698 cm^−1^, respectively, and the absorption band at 1513–1528 cm^−1^ was assigned to azomethine ν(C=N) stretching.

### 2.4. Spectroscopic Properties

**Figure 8:**
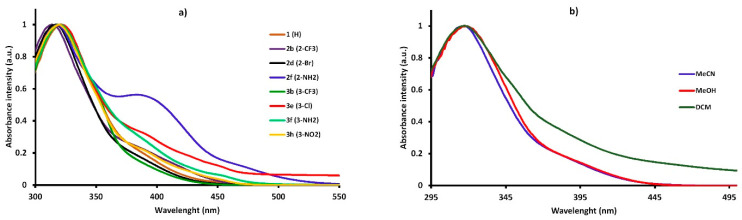
The normalized UV–Vis absorption of the synthesized 5-nitroisatin-based benzoylhydrazines (**a**) and the normalized UV–Vis absorption of 2-bromo-*N*′-[5-nitro-2-oxo-1,2-dihydro-3*H*-indol-3-ylidene]benzohydrazide (2d) in solvents of different polarity (**b**).

The spectral data of the synthesized compounds in different solvents are shown in Table 8. The study showed that new 5-nitroisatin-based benzoylhydrazines absorbed at around 320 nm and emitted (between 388 nm and 579 nm) over a wide spectral range with the extinction coefficients ranging from 1.92 × 10^4^ M^−1^ cm^−1^ to 2.80 × 10^4^ M^−1^ cm^−1^. The extinction coefficients did not achieve relatively high values. Additionally, no clear correlations between the solvent polarities and the molar extinction coefficients were found. The shape of the absorption spectra remained very similar to that of the parent compound (1) (R=H), and it was not significantly dependent on the electron-withdrawing or electron-releasing group in the different positions of the phenyl ring (Figure 8a). The position of the band of absorption did not depend on the polarity of the solvent (Figure 8b). The emission spectra were broad, with the single maximum of fluorescence that slightly shifted towards higher-wavelength values as the polarity of solvent increased.

## 3. Materials and Methods

### 3.1. Computational Methods

#### 3.1.1. The Docking Procedure

The structure of cyclin dependent kinase 2 (CDK2) was obtained from Brookhaven Protein Database PDB (CDK2—PDB ID 1E9H) [25]. The AutodockTools package [33] was used in all preliminary steps, including the identification and localization of the active site and the preparation of all structures of considered ligands and protein. In the case of each structure used during the molecular docking stage, all non-polar hydrogen atoms were removed. The localization of the center (x = 2.5, y = 35.0, z = 63.0) and the dimensions of the grid box have been adjusted to the size of the active site (16 × 14 × 22 Å). The docking procedure for all considered ligands was realized utilizing Autodock Vina package [34]. The value of exhaustiveness parameter was set at 20 for all realized simulations. In the case of each considered molecule, the docking procedure was repeated five times with a different value of the random seed.

#### 3.1.2. The Molecular Dynamics Simulations

The structure of the CDK2 protein was described using ff14sb [35] force-field parameters, while in the case of the inhibitor molecules, gaff force field parameters were used. In the case of isatin derivatives, the values of charges were estimated based on the Merz–Kollmann scheme using the RESP procedure at the HF/6-31G* level [36]. The complexes of the CDK2 protein with considered inhibitors were neutralized and immersed in the periodic cube box of TIP3P water molecules. Each system was heated to 300 K in the preliminary stage; the control of the temperature was realized utilizing Langevin thermostat [37]. The 80-nanosecond molecular dynamics simulations were realized with an applied shake algorithm and periodic boundary conditions. The structural analysis of considered systems, including the stability of interactions involved in the maintenance of complexes, were realized utilizing a VMD package [38]. The definition of hydrogen bonds was based on the following boundary criteria: distance D (donor)–A (acceptor) < 3.5 Å, distance H–A < 3 Å and angle D–H–A > 90°. The affinity of isatin derivatives towards the CDK2 active site was evaluated using the molecular mechanics Poisson–Boltzmann surface area (MMPBSA) [39]. The molecular dynamics simulations were realized using the AMBER 14 package [40].

#### 3.1.3. The Quantum Mechanics Calculations

The geometries of all the isatin derivatives were calculated using density functional theory (DFT) implemented in the Gaussian 09 package [41]. All calculations were realized using B3LYP functional at 6-311 + G (d,p) level of theory. The solvatation effects during calculations were included by applying a self-consistent reaction field (SCRF) approach [42] based on accurate numerical solutions of the Poisson–Boltzmann equation [43,44].

### 3.2. Materials

All reagents and solvents were purchased from Sigma-Aldrich (Poznań, Poland) and used without further purification. The highest (≥99%) purity of all used chemicals was required for spectroscopic studies.

### 3.3. Synthesis

The general procedure for the synthesis of the *N*′-[5-nitro-2-oxo-1,2-dihydro-3*H*-indol-3-ylidene]benzohydrazide.

Equimolar amounts of 5-nitroisatin (s1) (0.002 mol) and substituted benzolhydrazine (s2) (0.002 mol) were added to 96% ethanol (50 mL) containing 3 drops of glacial acetic acid. The mixture was heated under reflux for 5 h and then cooled to room temperature. The resulting solid was collected by filtration, washed with cold ethanol and recrystallized from ethanol to give the following compounds (1, 2b, 2d, 2f, 3b, 3e, 3f, and 3h).

The elemental analysis is as follows:

*N*′-[5-nitro-2-oxo-1,2-dihydro-3*H*-indol-3-ylidene]benzohydrazide (1) [30] Yellow solid, yield 85%, d.t. 338.4 °C, IR (ATR), cm^−1^: 3167, 1744, 1625, 1525, 1340. *Trans*-isomer (1): ^1^H NMR (DMSO-d_6_ from TMS) δ (ppm): 12.22 (s, 1H), 11.56 (s, 1H), 8.89 (s, 1H), 8.34 (dd,1H), 7.96 (m, 2H), 7.68 (m, 6H), 7.10 (d, *J* = 8.76, 1H). ^13^C NMR δ (ppm): 167.55, 165.44, 148.02, 142.31, 139.06, 132.96, 129.73, 129.18, 129.04, 128.06, 122.76, 115.78, 111.21. *Cis*-isomer (1′): ^1^H NMR (DMSO-d_6_ from TMS) δ (ppm): 13.74 (s, 1H), 12.03 (s, 1H), 8.31 (m, 2H), 7.92 (m, 2H), 7.68 (m, 6H), 7.18 (d, *J* = 8.24 Hz, 1H). ^13^C NMR δ (ppm): 163.87, 149.86, 143.41, 133.63, 132.18, 121.08, 116.29, 112.03. C_15_H_10_N_4_O_4_, Calcd. C, 58.07, H, 3.25, N, 18.06. Found C, 58.19, H, 3.30, N, 21.48.

2-trifluoromethyl-*N*′-[5-nitro-2-oxo-1,2-dihydro-3*H*-indol-3-ylidene]benzohydrazide (2b). Yellow solid, yield 87%, m.p. 267.4 °C, d.t. 310.3 °C, IR (ATR), cm^−1^: 3232, 1740, 1690, 1513, 1315. ^1^H NMR (DMSO-d_6_ from TMS) δ (ppm): 13.18 (s, 1H, 2b’), 12.88 (s, 1H, 2b), 11.96 (s, 1H, 2b’), 11.51 (s, 1H, 2b), 9.17 (bs, 1H), 8.85 (bs, 1H), 8.32 (m, 2H, 2b and 2b’), 7,87 (m, 8H, 2b and 2b’), 7.14 (d, *J* = 8.56 Hz, 1H, 2b’), 7.07 (d, *J* = 8.86 Hz, 1H, 2b). ^13^C NMR δ (ppm): 165.14, 163.46, 150.03, 148.30, 143.31, 142.42, 134.20, 132.92, 131.32, 129.38, 128.26, 126.91, 125.54, 122.82, 122.46, 120.75, 115.38, 112.05, 111.21. C_16_H_9_F_3_N_4_O_4_, Calcd. C, 50.80, H, 2.40, N, 14.81. Found C, 50.74, H, 2.34, N, 14,93.

2-bromo-*N*′-[5-nitro-2-oxo-1,2-dihydro-3*H*-indol-3-ylidene]benzohydrazide (2d) Yellow solid, yield 85%, m.p. 268.4 °C, d.t. 305.9 °C, IR (ATR), cm^−1^: 3303, 1749, 1698, 1495, 1339. ^1^H NMR (DMSO-d_6_ from TMS) δ (ppm): 13.16 (s, 1H, 2d’), 12.43 (s, 1H, 2d), 11.97 (s, 1H, 2d’), 11.52 (s, 1H, 2d), 9.06 (s, 1H, 2d), 8.34 (dd, 2H, 2d and 2d’), 7,79 (m, 2H, 2d and 2d’), 7.55 (m, 6H, 2d and 2d’), 7.15 (d, *J* = 8.72 Hz, 1H, 2d’), 7.07 (d, *J* = 8.72 Hz, 1H, 2d). ^13^C NMR δ (ppm): 165.20, 149.96, 148.24, 143.32, 142.43, 137.26, 135.64, 133.20, 132.24, 130.05, 129.31, 128.23, 122.49, 120.85, 119.68, 115.43, 112.06, 111.20. C_15_H_9_BrN_4_O_4_, Calcd. C, 46.29, H, 2.33, N, 14.40. Found C, 46.00, H, 2.43, N, 14.59.

2-Amino-*N*′-[5-nitro-2-oxo-1,2-dihydro-3*H*-indol-3-ylidene]benzohydrazide (2f) Dark orange solid, yield 91%, d.t. 320.1 °C, IR (ATR), cm^−1^: 3490, 3378, 3197, 1749, 1623, 1517, 1334. *Trans*-isomer (2f): ^1^H NMR (DMSO-d_6_ from TMS) δ (ppm): 11.52 (s, 1H), 8.83 (d, *J* = 2.16 Hz, 1H), 8.32 (m, 1H), 7.67 (m, 1H), 7.31 (m, 2H), 7.09 (d, *J* = 8.67 Hz, 1H), 6.87 (m, 2H), 6.67 (m, 2H). ^13^C NMR δ (ppm): 167.92, 165.56, 150.91, 149.56, 142.25, 138.28, 133.83, 130.01, 128.88, 122.36, 117.84, 115.92, 113.01, 111.11. *Cis*-isomer (2f’): ^1^H NMR (DMSO-d_6_ from TMS) δ (ppm): 13.64 (s, 1H), 11.97 (s, 1H), 8.30 (s, 1H), 8.27 (m, 1H), 7.45 (dd, 1H), 7.31 (m, 2H), 7.15 (d, *J* = 8.67 Hz, 1H), 6.87 (m, 2H), 6.67 (m, 2H). ^13^C NMR δ (ppm):165.21, 163.86, 151.90, 147.66, 143.33, 135.69, 134.37, 127.65, 127.60, 117.42, 115.66, 111.90, 111.60. C_15_H_11_N_5_O_4_, Calcd. C, 55.39, H, 3.41, N, 21.53. Found C, 55.20, H, 3.51, N, 21.62.

3-trifluoromethyl-*N*′-[5-nitro-2-oxo-1,2-dihydro-3*H*-indol-3-ylidene]benzohydrazide (3b) Yellow solid, yield 86%, m.p. 324.5 °C, d.t. 328.7 °C, IR (ATR), cm^−1^: 3197, 1713, 1685, 1526, 1341. *Cis*-isomer (3b’): ^1^H NMR (DMSO-d_6_ from TMS) δ (ppm): 13.68 (s, 1H), 12.01 (s, 1H), 8.30 (dd, 1H), 8.23 (s, 1H), 8.18 (s, 1H), 8.17 (d, *J* = 8.12 Hz, 1H), 8.09 (d, *J* = 7.80 Hz, 1H), 7.89 (t, 1H), 7.15 (d, *J* = 8.68 Hz, 1H). ^13^C NMR δ (ppm): 163.67, 148.12, 143.40, 137.28, 133.25, 132.10, 131.00, 130.37, 129.72, 128.22, 125.52, 124.99, 122.811, 120.84, 116.32, 112.09. Only the *cis*-isomer was observed. C_16_H_9_F_3_N_4_O_4_, Calcd. C, 50.80, H, 2.40, N, 14.81. Found C, 50.92, H, 2.36, N, 14,73.

3-chloro-*N*′-[5-nitro-2-oxo-1,2-dihydro-3*H*-indol-3-ylidene]benzohydrazide (3e). Yellow solid, yield 85%, d.t. 358.5 °C, IR (ATR), cm^−1^: 3189, 1679, 1676, 1527, 1340. *Trans*-isomer (3e): ^1^H NMR (DMSO-d_6_ from TMS) δ (ppm): 12.29 (s, 1H), 11.57 (s, 1H), 8.92 (s, 1H), 8.35 (dd, 1H), 8.00 (s, 1H), 7.91 (d, *J* = 7.8 Hz, 1H), 7.76 (m, 1H), 7.64 (t, 1H), 7.11 (d, *J* = 8.75, 1H). ^13^C NMR δ (ppm): 166.49, 165.34, 150.00, 142.33, 135.38, 133.71, 132.62, 130.99, 129.31, 129.00, 127.95, 122.90, 115.68, 111.25. *Cis*-isomer (3e’): ^1^H NMR (DMSO-d_6_ from TMS) δ (ppm): 13.67 (s, 1H), 12.04 (s, 1H), 8.31 (m, 1H), 8.30 (s, 1H), 7.93 (m, 1H), 7.86 (d, *J* = 7.84 Hz, 1H), 7.80 (m, 1H), 7.69 (t, 1H), 7.18 (d, *J* = 8.6 Hz, 1H). ^13^C NMR δ (ppm): 148.14, 134.39, 133.33, 131.70, 129.31, 128.24, 116.36, 112.10. C_15_H_9_ClN_4_O_4_, Calcd. C, 52.26, H, 2.63, N, 16.25. Found C, 52.16, H, 2.72, N, 16.30.

3-Amino-*N*′-[5-nitro-2-oxo-1,2-dihydro-3*H*-indol-3-ylidene]benzohydrazide (3f) Yellow solid, yield 92%, d.t. 327.4 °C, IR (ATR), cm^−1^: 3481, 3387, 3204, 1752, 1622, 1514, 1339. *Trans*-isomer (3f): ^1^H NMR (DMSO-d_6_ from TMS) δ (ppm): 12.02 (s, 1H), 11.53 (s, 1H), 8.80 (d, *J* = 1.96 Hz, 1H), 8.33 (dd, 1H), 7.24 (m, 1H), 7.18 (s, 1H), 7.09 (m, 2H), 6.85 (m, 1H), 5.48 (bs, 4H). ^13^C NMR δ (ppm): 163.86, 150.04, 149.54, 143.37, 139.06, 134.02, 129.51, 129.06, 122.64, 118.26, 115.89, 114.07, 111.76. *Cis*-isomer (3f’): ^1^H NMR (DMSO-d_6_ from TMS) δ (ppm): 13.65 (s, 1H), 11.99 (s, 1H), 8.30 (m, 2H), 7.24 (m, 1H), 7.21 (m, 3H), 6.98 (d, *J* = 8.24 Hz, 1H), 5.48 (bs, 4H). ^13^C NMR δ (ppm): 167.54, 164.07, 149.77, 147.51, 142.27, 136.55, 132.83, 130.12, 127.87, 121.19, 118.78, 116.17, 114.37, 112.99, 111.96. C_15_H_11_N_5_O_4_, Calcd. C, 55.39, H, 3.41, N, 21.53. Found C, 55.43, H, 3.40, N, 21.50.

3-nitro-*N*′-[5-nitro-2-oxo-1,2-dihydro-3*H*-indol-3-ylidene]benzohydrazide (3h). Yellow solid, yield 92%, m.p. 275.2 °C, d.t. 345.5 °C, IR (ATR), cm^−1^: 3197, 1747, 1684, 1528, 1341. *Trans*-isomer (3h): ^1^H NMR (DMSO-d_6_ from TMS) δ (ppm): 12.48 (s, 1H), 11.57 (s, 1H), 8.98 (s, 1H), 8.75 (s, 1H), 8.51 (m, 1H), 8.37 (m, 2H), 7.89 (t, 1H), 7.11 (d, *J* = 8.72 Hz, 1H). ^13^C NMR δ (ppm): 165.24, 150.06, 148.05, 142.37, 135.73, 134.81, 134.02, 130.65, 129.37, 127.17, 122.85, 124.13, 115.61, 111.27. *Cis-*isomer (3h’): ^1^H NMR (DMSO-d_6_ from TMS) δ (ppm): 13.73 (s, 1H), 12.04 (s, 1H), 8.68 (s, 1H), 8.54 (m, 1H), 8.33 (m, 2H), 8.29 (s, 1H), 7.18 (d, *J* = 8.72 Hz, 1H). ^13^C NMR δ (ppm): 163.65, 148.45, 148.20, 143.41, 134.81, 134.20, 133.63, 131.44, 129.37, 128.30, 123.12, 120.80, 116.42, 112.10. C_15_H_9_N_5_O_6_, Calcd. C, 50.71, H, 2.55, N, 19.71. Found C, 50.86, H, 2.52, N, 19,59.

### 3.4. Experimental Measurements

#### 3.4.1. NMR Measurements

The ^1^H NMR spectra were recorded using an Ascend III spectrometer operating at 400 MHz, Bruker. Chloroform was used as solvent and tetramethylsilane (TMS) as internal standard. Chemical shifts (*δ*) are reported in ppm relative to TMS and coupling constants (*J*) in Hz.

#### 3.4.2. Elemental Analysis Measurements

The elemental analysis was conducted with a Vario MACRO 11.45–0000, Elemental Analyser System GmbH, operating with the VARIOEL software (version 5.14.4.22).

#### 3.4.3. UV–VIS Measurements

The absorption and emission spectra were measured at room temperature in quartz cuvette (1 cm) using an Agilent Technology UV–Vis Cary 60 Spectrophotometer and a Hitachi F-7000 Spectrofluorometer, respectively.

#### 3.4.4. FTIR Measurements

The infrared spectra were recorded using reflectance spectroscopy measurements realized using PerkinElmer’s FTIR Spectrum Two, spectrophotometer equipped with diamond ATR in the range of 4000–400 cm^–1^.

#### 3.4.5. The Calorimetric Measurements

The thermal stability of 5-nitroisatin derivatives, considering the determination of the melting point (m.p.) and thermal decomposition temperature (d.t.), was realized using the DSC 6000 from PerkinElmer, Waltham, MA, USA. The calorimetric measurements were conducted with a heating rate of 10 K/min and 20 mL/min nitrogen flow to provide an inert atmosphere. The initial calibration the calorimeter was realized with indium and zinc standards; during measurements, standard aluminum pans were used.

## 4. Conclusions

The conducted research allowed for the development of a new group of 5-nitroisatin-based benzoylhydrazines, which show a significant affinity for the active site of CDK2. Based on the conducted analyses, it was possible to select groups of chemical substituents—the substitution of which, both in the isatin core and in the aromatic benzoylhydrazine ring, contributed to the improvement of the binding capacity of the considered derivatives. The conducted research allowed for the accurate characterization of the interactions responsible for maintaining the complexes of the considered inhibitors with the CDK2 enzyme. The most promising potential inhibitors, with the highest affinity values and forming the most stable complexes with the biological target, were directed to the experimental phase. A procedure for the synthesis of 5-nitroisatin derivatives was developed, and eight new compounds were obtained with a good yield. The structures of the newly synthesized compounds were confirmed using IR and NMR spectroscopy. It needs to be highlighted that the synthesized 5-nitroisatin-based benzoylhydrazines were present in the solution as *trans*- and *cis*-isomers. The absorption and fluorescence maxima were determined. It was found that the position of the bands of absorption and emission did not depend on the polarity of the solvent.

Based on the collected data—considering the binding capacity, the structural stability of the complexes and the molecular properties of the studied group of compounds—it can be concluded that the newly developed isatin derivatives can be used in the development of new anti-cancer therapies.

## Figures and Tables

**Table 1:** The values of binding affinity (∆G) of substituted isatin derivatives towards CDK2 active site. The reference value obtained for non-substituted isatin derivative is −8.9 (kcal/mol).

Name of Substituent	∆G Binding Affinity (kcal/mol)		
	R5	R6	R7
- I	−9.20	−8.80	−8.00
- Br	−8.80	−9.10	−8.10
- CH_3_	−9.40	−9.30	−8.60
- Cl	−8.80	−9.20	−8.30
- F	−9.20	−9.30	−8.60
- NO_2_	−9.50	---	−8.30
- OCH_3_	−9.10	−8.90	−8.20

**Table 2:** The values of biding affinity and inhibition constants (IC) of 5-nitroisatin-based benzoylhydrazines towards CDK2 active site.

Chemical Group	Name	Binding Affinity (kcal/mol)	IC [nM]	Name	Binding Affinity (kcal/mol)	IC [nM]	Name	Binding Affinity (kcal/mol)	IC [nM]
**-CH_3_**	2a	−9.60	91.9	3a	−10.00	46.8	4a	−9.57	96.6
**-CF_3_**	2b	−10.00	46.8	3b	−10.00	46.8	4b	−9.57	96.6
**-F**	2c	−9.70	77.6	3c	−9.80	65.5	4c	−9.50	108.7
**-Br**	2d	−9.70	77.6	3d	−9.73	73.8	4d	−9.50	108.7
**-Cl**	2e	−9.70	77.6	3e	−9.80	65.5	4e	−9.50	108.7
**-NH_2_**	2f	−9.70	77.6	3f	−9.70	77.6	4f	−9.40	128.7
**-N(CH_3_)_2_**	2g	−8.50	588.1	3g	−9.37	135.4	4g	−9.50	108.7
**-NO_2_**	2h	−9.23	171.5	3h	−9.77	68.9	4h	−9.50	108.7
**-OH**	2i	−9.47	114.4	3i	−9.40	128.7	4i	−9.40	128.7
**-OCH_3_**	2j	−9.27	160.3	3j	−9.47	114.4	4j	−9.37	135.4

**Table 3:** The values of hydrogen bond lengths [Å] involved in stabilization of 5-nitroisatin-based benzoylhydrazines complexes with the CDK2 active site.

Name	Hydrogen Bond Length [Å]						
	LYS33	GLU81	LEU83 H	LEU83 O	ASP145	ASP 86	LYS 89
1	2.28	2.23	1.83	2.91	2.71	---	---
2b	2.25	2.06	1.88	---	2.59	2.87	---
2c	2.36	2.05	1.83	---	2.53	---	---
2d	2.37	1.98	1.86	---	2.53	---	---
2e	2.34	2.03	1.84	---	2.55	---	---
2f	2.35	2.12	1.86	3.04	2.50	3.09	---
3a	2.28	2.19	1.81	2.95	2.64	---	---
3b	2.34	2.14	1.82	3.02	2.58	---	---
3c	2.31	2.23	1.83	2.89	2.66	---	---
3d	2.37	2.13	1.89	3.10	2.49	---	---
3e	2.26	2.20	1.84	2.93	2.64	---	---
3f	2.34	2.19	1.80	2.93	2.66	---	---
3h	2.33	2.35	1.81	2.54	2.81	---	2.38

**Table 4:** The averaged values of RMSD for ligands and CDK2 protein for all steps used during structural analysis.

Name	Ligand		CDK2	
	RMSD	SD	RMSD	SD
1	0.591	0.132	2.284	0.181
2b	0.698	0.146	2.745	0.419
2c	1.037	0.238	2.585	0.402
2d	0.525	0.101	2.316	0.209
2e	0.768	0.293	2.619	0.282
2f	0.689	0.177	2.265	0.245
3a	0.551	0.177	2.420	0.172
3b	0.708	0.155	2.618	0.164
3c	0.948	0.357	2.440	0.351
3d	0.603	0.204	2.504	0.400
3e	0.506	0.134	2.251	0.182
3f	0.475	0.085	2.431	0.263
3h	0.737	0.159	2.612	0.211

**Table 5:** The cumulative analysis of the length of the interactions identified in CDK2 complexes with selected 5-nitroisatin-based benzoylhydrazines. The distances presented in the table represent the middle values of intervals with a width of 0.2 Å.

Interactions	Population %								
	Σ	1.6 Å	1.8 Å	2 Å	2.2 Å	2.4 Å	2.6 Å	2.8 Å	3 Å
1									
Ligand (**H1**) … (**O**) GLU81	100.0	2.6	54.6	37.1	5.3	0.5	0.0	0.0	0.0
Ligand (**O1**) … (**HN**) LEU83	100.0	0.6	28.2	46.4	19.2	4.6	0.9	0.1	0.0
Ligand (**O**) … (**H**) LYS33	82.8	0.1	0.1	0.1	0.1	0.1	0.1	0.1	0.1
Ligand (**O**) … (**H**) ASP145	58.8	0.0	0.6	2.9	6.6	9.1	11.9	14.3	13.5
Ligand (**H5**) … (**O**) LEU83	19.62	0.0	0.1	0.9	0.6	0.9	2.5	3.7	10.9
2b									
Ligand (**H1**) … (**O**) GLU81	100.0	4.1	49.5	37.1	7.8	1.3	0.2	0.0	0.0
Ligand (**O2**) … (**HN**) LEU83	99.9	0.6	22.6	40.9	21.9	9.3	3.4	1.2	0.1
Ligand (**O**) … (**H**) LYS33	91.5	0.0	5.1	13.4	18.1	17.6	16.4	11.4	9.5
Ligand (**H5**) … (**O**) LEU83	85.5	0.8	16.7	29.3	12.9	5.2	4.1	6.0	10.4
Ligand (**F**) … (**O**) ILE10	64.2	0.0	0.0	0.0	0.0	0.0	2.8	20.0	41.3
Ligand (**O**) … (**H**) ASP145	91.0	0.1	3.0	16.8	21.2	15.9	14.6	10.5	8.9
2d									
Ligand (**H1**) … (**O**) GLU81	100.0	2.1	49.1	40.2	7.4	1.1	0.1	0.0	0.0
Ligand (**O1**) … (**HN**) LEU83	100.0	0.2	25.1	46.3	20.1	6.8	1.6	0.1	0.0
Ligand (**O**) … (**H**) LYS33	81.2	0.2	8.6	17.4	19.7	15.2	8.5	6.9	4.7
Ligand (**O**) … (**H**) ASP145	33.3	0.0	0.0	0.6	1.8	3.1	7.0	9.1	11.6
2f									
Ligand (**H1**) … (**O**) GLU81	100.0	2.1	48.4	41.6	6.8	0.8	0.1	0.1	0.0
Ligand (**O1**) … (**HN**) LEU83	100.0	0.6	23.0	48.1	21.3	5.5	1.3	0.3	0.0
Ligand (**O**) … (**H**) LYS33	83.6	0.1	9.7	15.8	18.3	14.3	11.3	8.1	6.1
Ligand (**O**) … (**H**) ASP145	65.6	0.0	2.4	7.9	10.8	15.8	12.4	8.4	8.1
3b									
Ligand (**H1**) … (**O**) GLU81	100.0	3.3	54.2	35.9	6.1	0.4	0.1	0.0	0.0
Ligand (**O1**) … (**HN**) LEU83	100.0	0.1	24.1	46.7	21.9	5.6	1.3	0.3	0.1
Ligand (**O**) … (**H**) LYS33	79.3	0.1	5.4	12.2	16.3	14.3	12.0	10.8	8.3
Ligand (**O**) … (**H**) ASP145	66.3	0.0	0.3	4.4	11.2	15.9	12.9	9.9	11.5
Ligand (**H5**) … (**O**) LEU83	11.4	0.0	0.0	0.1	0.4	0.6	0.8	2.6	7.0
3e									
Ligand (**H1**) … (**O**) GLU81	100.0	3.1	56.5	36.3	3.6	0.6	0.0	0.0	0.0
Ligand (**O1**) … (**HN**) LEU83	100.0	0.4	26.7	48.1	19.4	4.8	0.5	0.0	0.0
Ligand (**O**) … (**H**) LYS33	79.8	0.1	7.2	20.8	19.7	13.9	7.3	5.9	4.9
Ligand (**O**) … (**H**) ASP145	86.8	0.0	1.4	11.3	19.2	20.8	16.6	9.9	7.6
3f									
Ligand (**H3**) … (**O**) GLU81	100.0	1.9	49.3	39.4	8.4	0.9	0.1	0.0	0.0
Ligand (**O2**) … (**HN**) LEU83	100.0	0.1	12.1	39.1	31.1	14.4	2.6	0.8	0.0
Ligand (**O**) … (**H**) LYS33	71.3	0.1	3.9	10.4	14.8	12.6	11.5	10.8	7.1
Ligand (**O**) … (**H**) ASP145	55.5	0.0	0.8	4.9	7.9	9.1	8.6	11.8	12.5
Ligand (**H5**) … (**O**) LEU83	98.9	0.3	18.3	33.4	25.9	12.2	5.5	2.3	1.1
3h									
Ligand (**H1**) … (**O**) GLU81	100.0	1.9	48.0	42.4	7.1	0.6	0.1	0.0	0.0
Ligand (**O1**) … (**HN**) LEU83	100.0	0.5	22.3	47.9	21.8	5.8	1.6	0.1	0.1
Ligand (**O**) … (**H**) LYS33	84.4	0.2	12.8	19.9	16.4	13.5	10.8	5.9	4.8
Ligand (**O**) … (**H**) ASP145	67.6	0.0	2.6	4.4	8.2	10.6	11.1	14.6	16.2
Ligand (**H5**) … (**O**) LEU83	15.4	0.0	0.1	0.8	0.8	0.9	2.0	3.6	7.3

**Table 6:** Energies of *cis-* and *trans*-isomers of 5-nitroisatin-based benzoylhydrazines estimated in DMSO at the B3LYP/6-311 ++ G(d,p) level of theory. **∆E*_CIS-TRANS_*** represents the difference between *cis-* and *trans-*isomers (**∆E*_CIS-TRANS_*** = **E_CIS_** − **E_TRANS_**).

Name	E_CIS_	E_TRANS_	∆E*_CIS-TRANS_*	∆E*_CIS-TRANS_*
	[Hartree]	[Hartree]	[Hartree]	(kcal/mol)
1	−1097.75447	−1097.74754	−0.0069268	−4.347
2b	−1434.89555	−1434.88862	−0.0069287	−4.348
2d	−3671.28947	−3671.28262	−0.0068435	−4.294
2f	−1153.13766	−1153.13059	−0.0070714	−4.437
3b	−1434.90262	−1434.89550	−0.0071178	−4.466
3e	−1557.37551	−1557.36858	−0.0069289	−4.348
3f	−1153.13538	−1153.12863	−0.0067527	−4.237
3h	−1302.31909	−1302.31183	−0.0072546	−4.552

**Table 7:** Characteristic band-strength values of the synthesized 5-nitroisatin-based benzoylhydrazines.

Compound	ν(–NH_2_)		ν(N–H)	ν(C=O)		ν(C=N)	ν(N=O)
	ν_asym_	ν_sym_		Lactone	Hydrazide		
1	-	-	3167	1744	1625	1525	1340
2b	-	-	3232	1740	1690	1513	1315
2d	-	-	3303	1749	1698	1495	1339
2f	3490	3378	3197	1749	1623	1517	1334
3b	-	-	3197	1713	1685	1526	1341
3e	-	-	3189	1679	1676	1527	1340
3f	3481	3387	3204	1752	1622	1514	1339
3h	-	-	3197	1747	1684	1528	1341

**Table 8:** Spectroscopic properties of the synthesized 5-nitroisatin-based benzoylhydrazines in solvents of different polarity.

No.	Substituent	Solvent	*λ_ab_* (nm)	*λ_fl_* (nm)	*ε* (×10^4^, M^−1^ ·cm^−1^)	Stokes Shift (cm^−1^)
1	H	DCM MeOH MeCN	320 323 322	522 573 542	a 3.19 2.28	12,092 13,507 12,605
2b	2-CF_3_	DCM MeOH MeCN	315 315 314	517 575 538	a 2.72 2.27	12,403 14,354 13,259
2d	2-Br	DCM MeOH MeCN	317 319 318	523 574 539	a 2.51 2.16	12,425 13,926 12,893
2f	2-NH_2_	DCM MeOH MeCN	320 320 318	437 417 382	a 1.92 1.98	8366 13,926 5268
3b	3-CF_3_	DCM MeOH MeCN	319 319 318	522 579 540	a 2.52 2.80	12,190 14,076 12,928
3e	3-Cl	DCM MeOH MeCN	319 325 320	530 556 514	a 1.32 1.18	12,480 12,783 11,794
3f	3-NH_2_	DCM MeOH MeCN	321 321 320	435 388 527	a 2.38 2.27	8164 5379 12,274
3h	3-NO_2_	DCM MeOH MeCN	319 321 320	522 577 547	a 2.10 1.88	12,190 13,821 12,968

a—compounds partially insoluble in DCM.

## Data Availability

Data is contained within the article or supplementary material.

## Supplementary Materials

The following supporting information can be downloaded at: https://www.mdpi.com/article/10.3390/ijms23148046/s1.
