# Supplementary material for: Designing and Synthesis of New Isatin Derivatives as Potential CDK2 Inhibitors
Source: Int J Mol Sci. 2022 Jul 21;23(14):8046. doi: 10.3390/ijms23148046 (PMC9316372; doi:10.3390/ijms23148046)
Supplement: Supplementary file 1 [file ijms-23-08046-s001.zip › ijms-1814390-supplementary.pdf]

Designing and synthesis of new isatin derivatives as potential CDK2 inhibitors

Przemysław Czeleń [1*], Agnieszka Skotnicka [2] and Beata Szefler [1]

[1]Department of Physical Chemistry, Faculty of Pharmacy, Collegium Medicum, Nicolaus Copernicus University, Kurpinskiego 5, 85-096 Bydgoszcz, Poland
[2]Faculty of Chemical Technology and Engineering, Bydgoszcz University of Science and Technology, Seminaryjna 3, 85-326 Bydgoszcz, Poland

Supplementary materials:

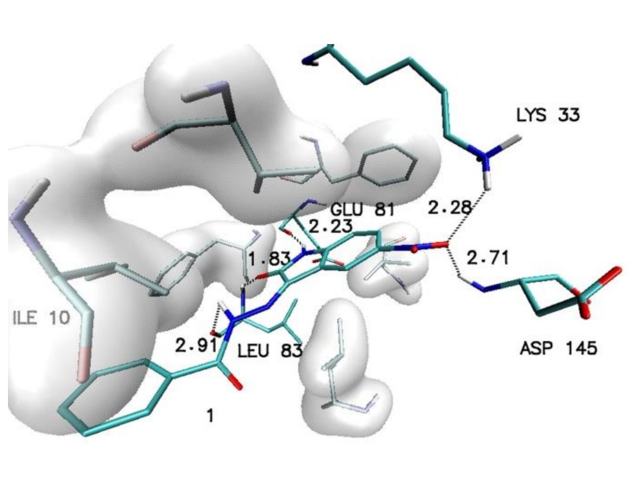

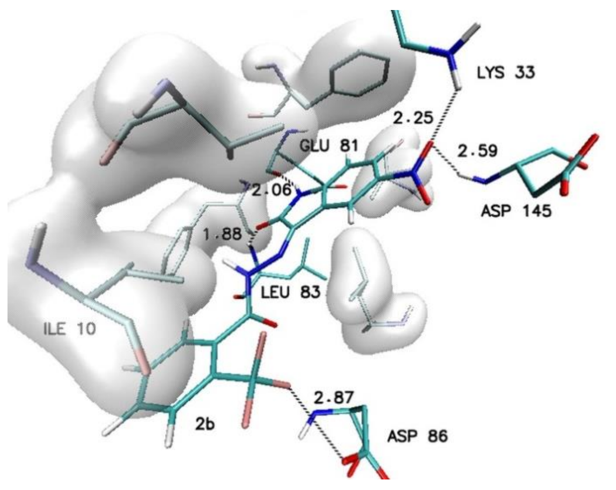

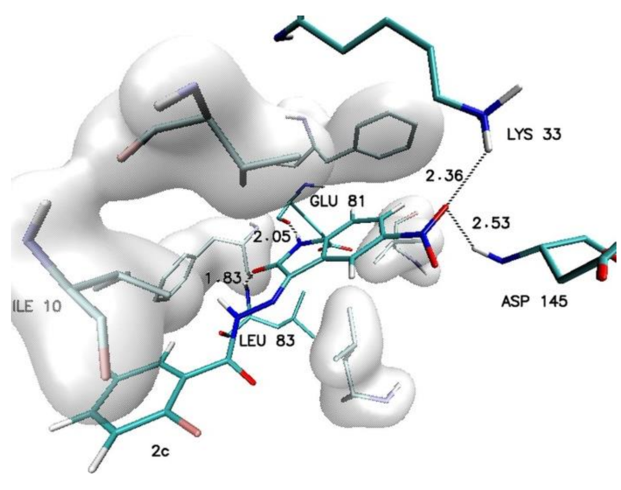

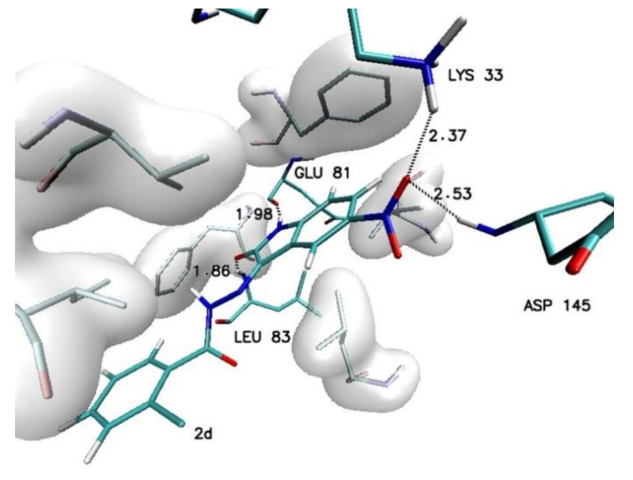

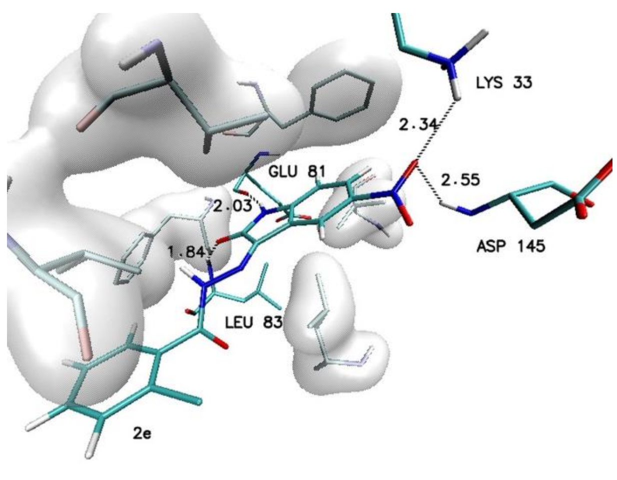

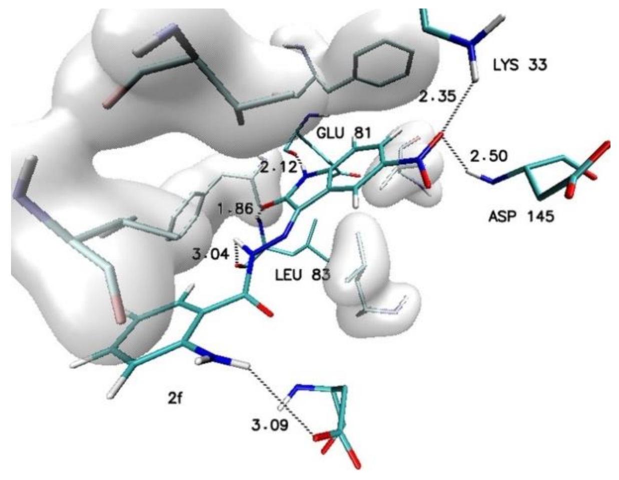

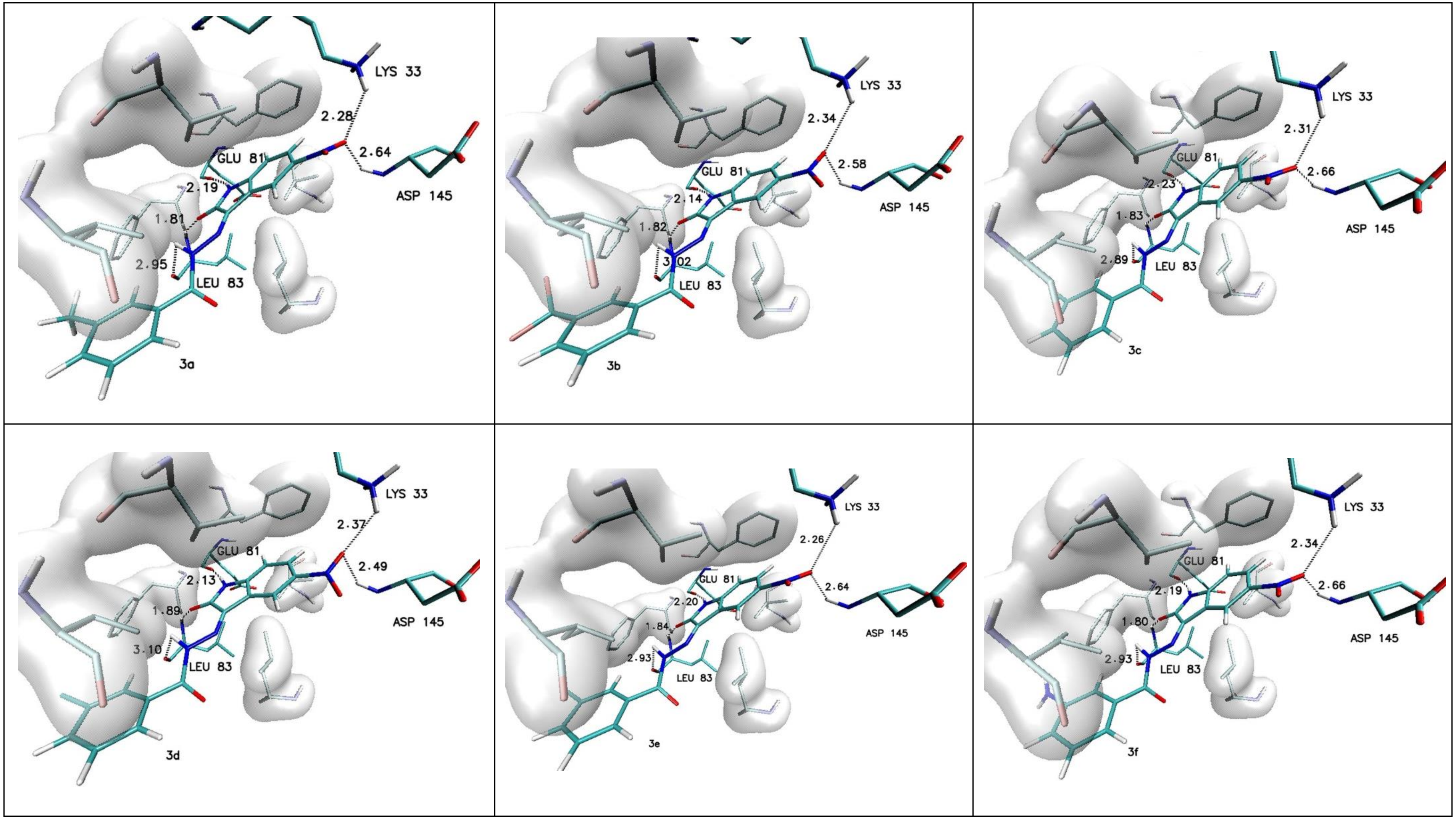
LYS 33
2.28
2.64
ASP 145
GLU 81
2.19
1.81
2.95
LEU 83
3a
LYS 33
2.34
2.58
ASP 145
GLU 81
2.14
1.82
3.02
LEU 83
3b
LYS 33
2.31
2.66
ASP 145
GLU 81
2.23
1.83
2.89
LEU 83
3c
LYS 33
2.37
2.49
ASP 145
GLU 81
2.13
1.89
3.10
LEU 83
3d
LYS 33
2.26
2.64
ASP 145
GLU 81
2.20
1.84
2.93
LEU 83
3e
LYS 33
2.34
2.66
ASP 145
GLU 81
2.19
1.80
2.93
LEU 83
3f

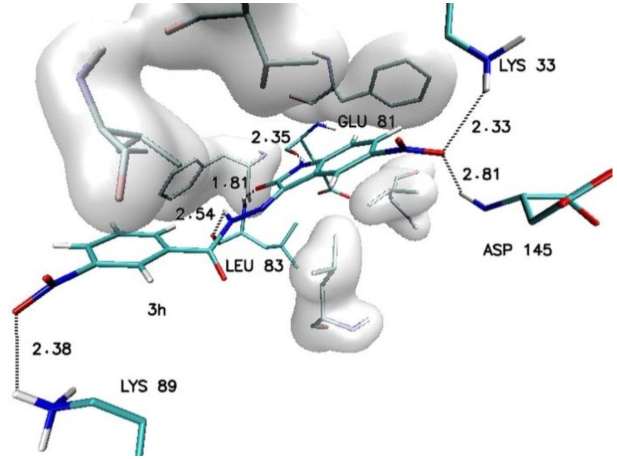		

Figure S1. The graphic representation of interactions involved in stabilization of CDK2 complex with 5-nitroisatin-based benzoylhydrazines analyzed during molecular dynamics simulations.

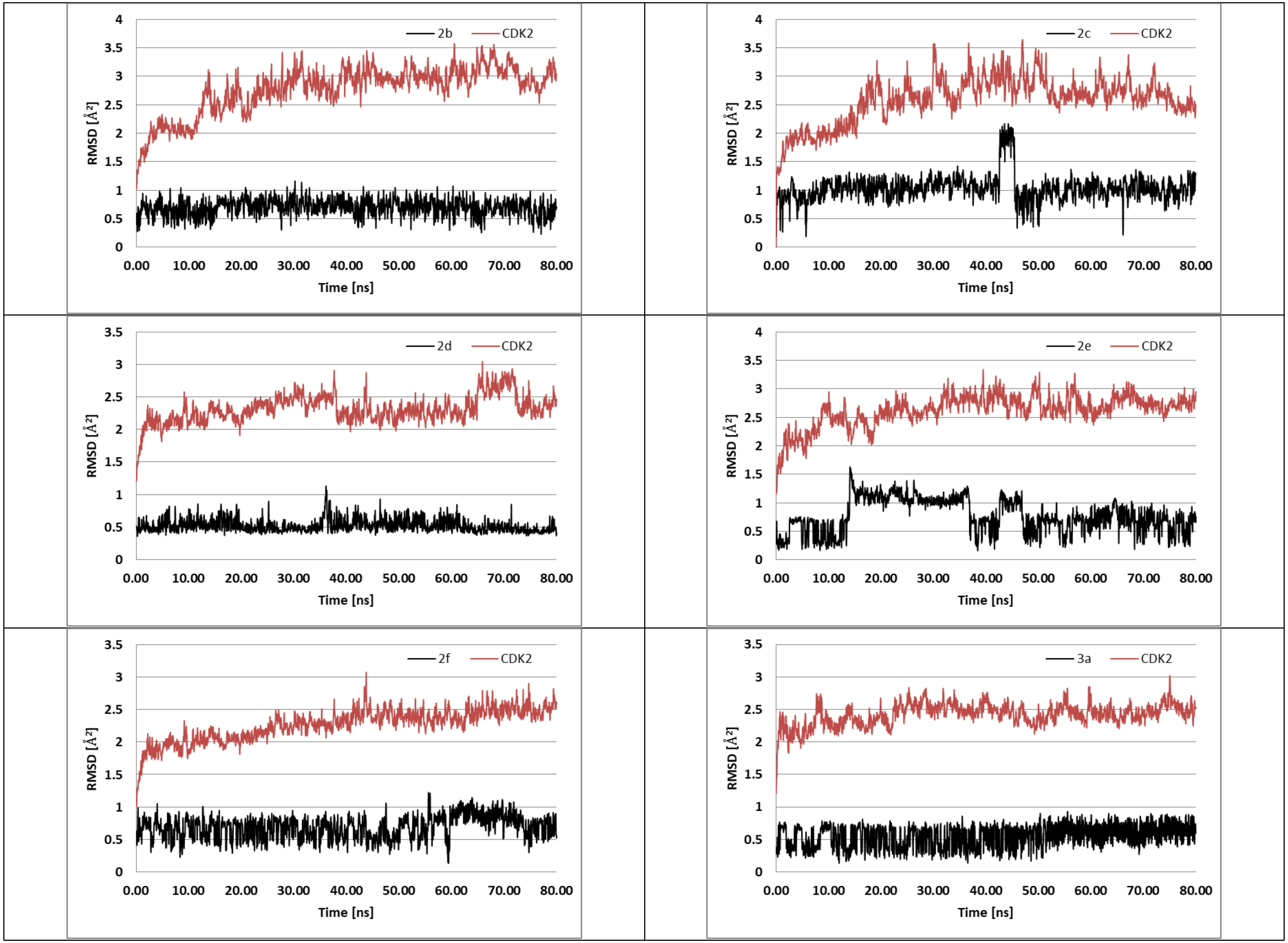

2b
CDK2
RMSD [Å²]
Time [ns]
2c
CDK2
RMSD [Å²]
Time [ns]
2d
CDK2
RMSD [Å²]
Time [ns]
2e
CDK2
RMSD [Å²]
Time [ns]
2f
CDK2
RMSD [Å²]
Time [ns]
3a
CDK2
RMSD [Å²]
Time [ns]
0.00 10.00 20.00 30.00 40.00 50.00 60.00 70.00 80.00

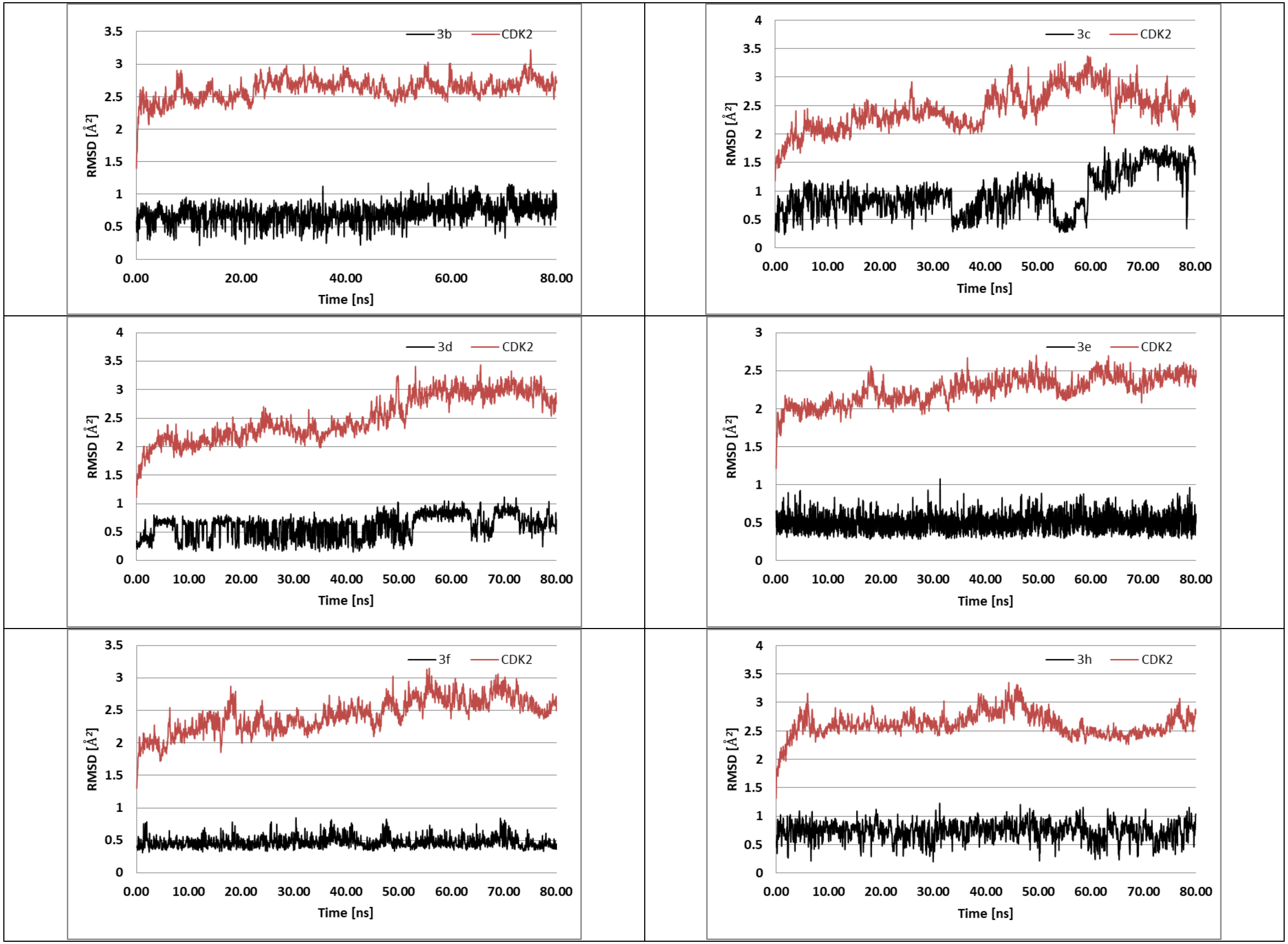
3b
CDK2
RMSD [Å²]
Time [ns]
3c
CDK2
RMSD [Å²]
Time [ns]
3d
CDK2
RMSD [Å²]
Time [ns]
3e
CDK2
RMSD [Å²]
Time [ns]
3f
CDK2
RMSD [Å²]
Time [ns]
3h
CDK2
RMSD [Å²]
Time [ns]

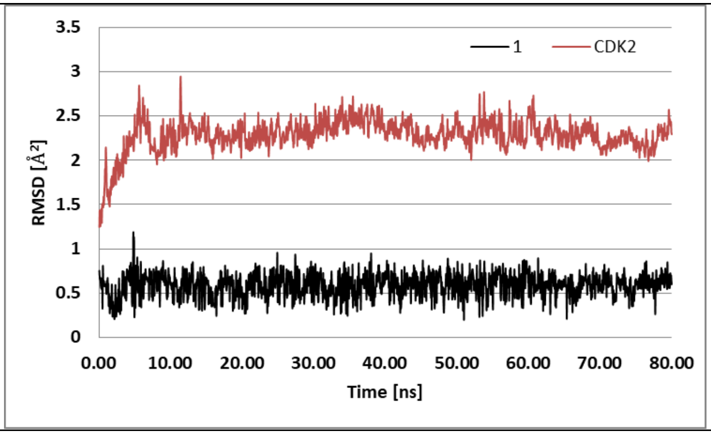

Figure S2. Distributions of RMSD values. Black distributions refer to ligand molecules while brown distributions refer to CDK-2 protein.

Table S1. The cumulative analysis of the length of the interactions identified in CDK2 complexes with 5-nitroisatin-based benzoylhydrazines. The distances presented in the table represent middle values of intervals with a width of 0.2 Å.

INTERACTIONS	Population %								
	Σ	1.6	1.8	2	2.2	2.4	2.6	2.8	3
1									
Ligand (**H1**) … (**O**) GLU 81	100.0	2.6	54.6	37.1	5.3	0.5	0.0	0.0	0.0
Ligand (**O1**) … (**HN**) LEU 83	100.0	0.6	28.2	46.4	19.2	4.6	0.9	0.1	0.0
Ligand (**O**) … (**H**) LYS 33	82.8	0.1	0.1	0.1	0.1	0.1	0.1	0.1	0.1
Ligand (**O**) ... (**H**) ASP 145	58.8	0.0	0.6	2.9	6.6	9.1	11.9	14.3	13.5
Ligand (**H5**) … (**O**) LEU 83	19.62	0.0	0.1	0.9	0.6	0.9	2.5	3.7	10.9
2b									
Ligand (**H1**) … (**O**) GLU 81	100.0	4.1	49.5	37.1	7.8	1.3	0.2	0.0	0.0
Ligand (**O2**) … (**H**N) LEU 83	99.9	0.6	22.6	40.9	21.9	9.3	3.4	1.2	0.1
Ligand (**O**) … (**H**) LYS 33	91.5	0.0	5.1	13.4	18.1	17.6	16.4	11.4	9.5
Ligand (**H5**) … (**O**) LEU 83	85.5	0.8	16.7	29.3	12.9	5.2	4.1	6.0	10.4
Ligand (**F**) ... (**O**) ILE 10	64.2	0.0	0.0	0.0	0.0	0.0	2.8	20.0	41.3
Ligand (**O**) ... (**H**) ASP 145	91.0	0.1	3.0	16.8	21.2	15.9	14.6	10.5	8.9
2c									
Ligand (**H1**) … (**O**) GLU 81	100.0	2.4	39.2	40.6	16.8	0.9	0.1	0.0	0.0
Ligand (**O1**) … (**H**N) LEU 83	99.9	0.5	13.3	39.6	28.7	11.9	4.3	1.6	0.2
Ligand (**O**) … (**H**) LYS 33	61.4	0.1	4.1	11.1	12.3	10.6	9.6	7.9	5.8
Ligand (**O**) ... (**H**) ASP 145	36.8	0.0	0.4	4.0	5.6	8.8	4.7	7.0	6.3
Ligand (**F**) ... (**O**) ILE 10	28.4	0.0	0.0	0.0	0.0	0.0	0.7	8.8	19.0
2d									
Ligand (**H1**) … (**O**) GLU 81	100.0	2.1	49.1	40.2	7.4	1.1	0.1	0.0	0.0
Ligand (**O1**) … (**H**N) LEU 83	100.0	0.2	25.1	46.3	20.1	6.8	1.6	0.1	0.0
Ligand (**O**) … (**H**) LYS 33	81.2	0.2	8.6	17.4	19.7	15.2	8.5	6.9	4.7
Ligand (**O**) ... (**H**) ASP 145	33.3	0.0	0.0	0.6	1.8	3.1	7.0	9.1	11.6
2e									

Ligand (**H1**) … (**O**) GLU 81	100.0	2.3	52.2	39.5	5.8	0.3	0.1	0.0	
Ligand (**O1**) … (**H**N) LEU 83	100.0	0.2	22.1	48.1	22.7	5.4	1.1	0.4	0.1
Ligand (**O**) … (**H**) LYS 33	51.1	0.1	3.9	8.7	9.5	8.6	7.7	6.8	5.9
Ligand (**O**) ... (**H**) ASP 145	54.3	0.0	0.1	2.9	6.3	7.7	10.4	12.6	14.2
2f									
Ligand (**H1**) … (**O**) GLU 81	100.0	2.1	48.4	41.6	6.8	0.8	0.1	0.1	0.0
Ligand (**O1**) … (**H**N) LEU 83	100.0	0.6	23.0	48.1	21.3	5.5	1.3	0.3	0.0
Ligand (**O**) … (**H**) LYS 33	83.6	0.1	9.7	15.8	18.3	14.3	11.3	8.1	6.1
Ligand (**O**) ... (H) ASP 145	65.6	0.0	2.4	7.9	10.8	15.8	12.4	8.4	8.1
3a									
Ligand (**H1**) … (**O**) GLU 81	100.0	2.3	44.2	45.9	7.1	0.4	0.1	0.0	0.0
Ligand (**O1**) … (**H**N) LEU 83	100.0	0.1	20.1	44.4	26.2	5.6	3.3	0.3	0.1
Ligand (**O**) … (**H**) LYS 33	57.3	0.1	2.4	7.2	9.3	9.3	10.0	10.8	8.3
Ligand (**O**) ... (**H**) ASP 145	55.3	0.0	0.3	3.4	7.2	9.9	12.9	9.9	11.5
Ligand (**H5**) … (**O**) LEU 83	11.4	0.0	0.0	0.1	0.4	0.6	0.8	2.6	7.0
3b									
Ligand (**H1**) … (**O**) GLU 81	100.0	3.3	54.2	35.9	6.1	0.4	0.1	0.0	0.0
Ligand (**O1**) … (**H**N) LEU 83	100.0	0.1	24.1	46.7	21.9	5.6	1.3	0.3	0.1
Ligand (**O**) … (**H**) LYS 33	79.3	0.1	5.4	12.2	16.3	14.3	12.0	10.8	8.3
Ligand (**O**) ... (H) ASP 145	66.3	0.0	0.3	4.4	11.2	15.9	12.9	9.9	11.5
Ligand (**H5**) … (**O**) LEU 83	11.4	0.0	0.0	0.1	0.4	0.6	0.8	2.6	7.0
3c									
Ligand (**H1**) … (**O**) GLU 81	100.0	1.9	37.1	40.9	18.7	1.2	0.2	0.0	0.0
Ligand (**O1**) … (**H**N) LEU 83	100.0	0.6	22.1	43.6	27.8	4.4	1.0	0.4	0.0
Ligand (**O**) … (**H**) LYS 33	44.2	0.0	1.8	5.8	6.9	7.4	7.9	7.1	7.4
3d									
Ligand (**H1**) … (**O**) GLU 81	99.9	3.1	56.8	34.2	5.6	0.3	0.0	0.0	0.0
Ligand (**O1**) … (**H**N) LEU 83	99.9	0.4	23.2	45.1	23.1	6.3	1.4	0.4	0.0
Ligand (**O**) … (**H**) LYS 33	72.3	0.1	5.1	13.4	13.7	10.6	13.5	8.6	7.3
Ligand (**O**) ... (**H**) ASP 145	46.2	0.0	0.7	3.2	5.9	7.7	8.4	9.1	11.3

3e									
Ligand (**H1**) … (**O**) GLU 81	100.0	3.1	56.5	36.3	3.6	0.6	0.0	0.0	0.0
Ligand (**O1**) … (**H**N) LEU 83	100.0	0.4	26.7	48.1	19.4	4.8	0.5	0.0	0.0
Ligand (**O**) … (**H**) LYS 33	79.8	0.1	7.2	20.8	19.7	13.9	7.3	5.9	4.9
Ligand (**O**) ... (**H**) ASP 145	86.8	0.0	1.4	11.3	19.2	20.8	16.6	9.9	7.6
3f									
Ligand (**H3**) … (**O**) GLU 81	100.0	1.9	49.3	39.4	8.4	0.9	0.1	0.0	0.0
Ligand (**O2**) … (**H**N) LEU 83	100.0	0.1	12.1	39.1	31.1	14.4	2.6	0.8	0.0
Ligand (**O**) … (**H**) LYS 33	71.3	0.1	3.9	10.4	14.8	12.6	11.5	10.8	7.1
Ligand (**O**) ... (**H**) ASP 145	55.5	0.0	0.8	4.9	7.9	9.1	8.6	11.8	12.5
Ligand (**H5**) … (**O**) LEU 83	98.9	0.3	18.3	33.4	25.9	12.2	5.5	2.3	1.1
3h									
Ligand (**H1**) … (**O**) GLU 81	100.0	1.9	48.0	42.4	7.1	0.6	0.1		
Ligand (**O1**) … (**H**N) LEU 83	100.0	0.5	22.3	47.9	21.8	5.8	1.6	0.1	0.1
Ligand (**O**) … (**H**) LYS 33	84.4	0.2	12.8	19.9	16.4	13.5	10.8	5.9	4.8
Ligand (**O**) ... (**H**) ASP 145	67.6	0.0	2.6	4.4	8.2	10.6	11.1	14.6	16.2
Ligand (**H5**) … (**O**) LEU 83	15.4	0.0	0.1	0.8	0.8	0.9	2.0	3.6	7.3
